# Yb^3+^-Doped Titanate–Germanate Glasses for Near-IR Luminescence Applications: Synthesis, Characterization, and the Influence of TiO_2_ Concentration

**DOI:** 10.3390/ma17235874

**Published:** 2024-11-29

**Authors:** Karolina Kowalska, Joanna Pisarska, Wojciech A. Pisarski

**Affiliations:** Institute of Chemistry, University of Silesia, Szkolna 9 Street, 40-007 Katowice, Poland; wojciech.pisarski@us.edu.pl

**Keywords:** inorganic glasses, TiO_2_, lanthanide ions, near-IR luminescence, photonics applications

## Abstract

In the framework of luminescent rare-earth-doped glasses for near-infrared applications, TiO_2_-containing inorganic glasses have been recently demonstrated to be a promising alternative to commercially used high-phonon SiO_2_-based glasses. This study investigates the effect of TiO_2_ concentration on the near-infrared spectroscopic properties of Yb^3+^ ions in multicomponent titanate–germanate glasses. A series of glass samples in the xTiO_2_-(60−x)GeO_2_-BaO-Ga_2_O_3_-Yb_2_O_3_ system (x ranging from 0 to 50 mol%) were synthesized using the melt-quenching technique. X-ray diffraction analysis confirmed the fully amorphous nature of the fabricated titanate–germanate samples. Fundamental spectroscopic properties of Yb^3+^-doped titanate–germanate system consisting of absorption spectra, near-IR emission spectra, and luminescence decay curves have been determined based on measurement using optical spectroscopy. The intensity of the emission band at 1 µm due to the ^2^F_5/2_ → ^2^F_7/2_ laser transition of Yb^3+^ ions increases by over 2.3-fold (TiO_2_ as the network former) compared to a barium gallo-germanate sample without TiO_2_. Our previous studies indicate that Yb^3+^-doped titanate–germanate glass is a promising optical material and could be successfully applied to laser technology.

## 1. Introduction

Glass is one of the most frequently analyzed and most essential materials for understanding structural, thermal, and optical properties. Recently, a lot of interest has been directed towards glasses emitting efficient radiation in the near-infrared range (NIR) [1,2,3,4,5]. In particular, various glass matrices [6,7,8,9,10] activated by rare-earth ions are still tested in order to obtain excellent materials with enhanced luminescent bands and longer lifetimes.

In the case of inorganic glasses containing TiO_2_ (TiO_2_ < 25%mol), most of the systems possess crystalline phases mainly because of the different titanates, which makes them unsuitable for fiber applications [11,12]. Our TiO_2_-GeO_2_-BaO-Ga_2_O_3_ host is the first example of a fully amorphous glass with relatively high TiO_2_ content (up to 50 mol%). It has good thermal stability parameters, required in modern optical fiber technology, and spectacular spectroscopic and laser parameters for the main laser transition of rare-earth ions [13,14,15,16,17]. The key factor to receiving such unique near-infrared luminescence features is a change in the glass network, which leads to a change in the physicochemical properties of the germanate glasses. TiO_2_ plays the key role as a glass modifier (TiO_2_ < 30 molar%) or glass former (TiO_2_ > 30 molar%). It has been experimentally proven that TiO_2_ participates in forming the glass network with the stretching vibration of Ti-O in the TiO_6_ unit [18]. On the other hand, our previous measurements of near-IR emission spectra and their luminescence decays clearly indicated that the spectroscopic properties of systems with various GeO_2_:TiO_2_ molar ratios strongly depend on the ionic radius of rare-earth ions. Indeed, low (TiO_2_ < 30 molar%) and high (TiO_2_ > 30 molar%) concentrations of titanium dioxide favored the near-IR luminescence of rare-earth ions located at the beginning, Pr^3+^ (1.00 Å) or Nd^3+^ (0.98 Å), and the end, Er^3+ (^0.89 Å) or Tm^3+^ (0.88 Å), of the lanthanide series [19]. All these characteristics indicate that this glass can be used as a material for photonics applications. It is well known that the ionic radius of Ti^3+^ is equal to 0.67 Å. Notably, the ionic radius of Yb^3+^ (0.87 Å) is the smallest of the lanthanide series [20] and comparable to the ionic radius of Er^3+^ and Tm^3+^ ions. For this reason, an interesting cognitive aspect in a further part of this study will be to analyze the effect of TiO_2_ concentration on changes in the spectroscopic properties of Yb^3+^ ions in barium gallo-germanate glass.

In general, ytterbium (Yb^3+^) has been of great interest due to its simple energy level scheme, which is composed of a single optically excited level (^2^F_5/2_). Yb^3+^ is an important luminescent element for various photonics applications such as infrared lasers and amplifiers with wavelengths near 1 µm [21]. In recent years, Yb^3+^ ions have been successfully incorporated into several glasses [22,23,24,25,26] and crystals [27,28,29,30] to obtain luminescent materials. Concerning the glasses, Koch et al. [31] in 1997 presented an efficient room-temperature Yb:glass laser pumped by a 946 nm Nd:YAG laser with near 48% slope efficiency. In Petrov et al. [32], Yb^3+^-doped fluoride phosphate glass was pumped by a Ti:sapphire laser based on Kerr-lens mode-locked technology. In 2024, Zhu et al. [33] demonstrated the first diode-pumped wavelength-tunable Yb^3+^-doped low-silica calcium aluminosilicate (LSCAS) glass lasers. Recently, interesting results were presented by the Petit group [34], which developed an alternative material for the future rare-earth metal shortage. In this case, the novel Yb^3+^-doped tellurite glasses were prepared using a standard melting process. These authors presented that the precipitation of crystals in low-Yb^3+^-concentrated glasses in the TeO_2_–ZnO–Bi_2_O_3_ network can be used to increase the intensity of the emission at ∼1 µm of Yb^3+^ ion emission to the level of highly Yb^3+^-concentrated glasses. The spectroscopic features of ytterbium ions strictly depend on the chemical composition of the glass. This finding was also clearly confirmed by a study conducted by Zhang et al. [35]. Based on the crystal-field and asymmetry of Yb^3+^ theories, the Stark splitting of Yb^3+^ was the highest in germanate glass and the lowest in phosphate glass. To the best of our knowledge, no systematic studies of any luminescence properties of the germanate-based glass host with different TiO_2_:GeO_2_ ratios in order to achieve enhancement of the near-IR emission band due to the ^2^F_5/2_ → ^2^F_7/2_ transition have ever been presented. The selected barium gallo-germanate glass in our study is a good candidate to build an optical device due to its good mechanical properties, high rare-earth solubility, comparatively low phonon energy, and wide emission spectra in the near-IR region.

This paper presents Yb^3+^-doped GeO_2_–BaO–Ga_2_O_3_ and TiO_2_–GeO_2_–BaO–Ga_2_O_3_ glasses as the optical material in terms of their near-infrared photonic applications. The glasses were synthesized by the melt-quenching technique, followed by an experimental investigation encompassing measurements by X-ray diffraction (XRD), optical absorption, and luminescence spectroscopy. We discuss the effect of the chemical composition of glass on the near-infrared luminescence features of Yb^3+^ ions, proving the importance of the dual role of titanium dioxide in the glass network. At the end of this study, the role of TiO_2_ in the materials science field and potential applications of the fabricated Yb^3+^-doped titanate–germanate glass are discussed below. It was found that the spectroscopic properties of Yb^3+^-doped barium gallo-germanate glass depends on TiO_2_ concentration, which confirms the enhanced near-IR emission band at 1 µm due to the ^2^F_5/2_ → ^2^F_7/2_ laser transition.

## 2. Materials and Methods

### 2.1. Yb^3+^-Doped Titanate–Germanate Glasses: Synthesis

A series of low-phonon titanate–germanate glasses emitting radiation in the near-infrared range were prepared using a high-temperature melt-quenching method. This experiment mainly focused on studying the effect of TiO_2_ content on the optical properties of the barium gallo-germanate (60GeO_2_-30BaO-9.5Ga_2_O_3_-0.5Yb_2_O_3_) glass system with the following chemical compositions: xTiO_2_-(60-x)GeO_2_-30BaO-9.5Ga_2_O_3_-0.5Yb_2_O_3_ (where x = 10, 20, 30, 40, 45, and 50), respectively. The concentrations of the components are given in molar %. For better clarity, the chemical compositions of the studied barium gallo-germanate samples with various GeO_2_:TiO_2_ molar ratios are shown in Table 1.

Samples were prepared under rigorous technological conditions in a special glove-box, in a protective atmosphere of dried argon of high purity. The restrictive procedure of synthesis has also been implemented for rare-earth-doped titanate–germanate glasses [14,15,36], which confirmed the obtained extremely low IR absorption coefficients. High-purity raw materials were GeO_2_ (99.99%), TiO_2_ (99.995%, rutile), BaO (99.99%), Ga_2_O_3_ (99.99%), and Yb_2_O_3_ (99.99%) and they were all purchased from Sigma-Aldrich (St. Louis, MO, USA). The synthesis of titanate–germanate glasses can be summarized by five different steps: (1) the appropriate amounts of all components of 5 g were mixed homogeneously together in an agate mortar, (2) samples were melted in a high-temperature furnace (FCF 4/170 M, produced by Czylok, Poland) at 1250 °C for 0.45 h in corundum crucibles (Łukasiewicz Research Network, Institute of Ceramics and Building Materials, Cracow, Poland), (3) samples were quenched and annealed below the glass transition temperature T_g_ to eliminate internal mechanical stresses, (4) samples were polished (grinding/polishing machine Struers Labopol-2), and (5) glass samples were prepared for optical measurements.

### 2.2. Measurements and Characterization

The structural characterization of the fabricated samples was provided by X-ray diffraction (XRD) analysis using an X’Pert Pro X-ray diffractometer supplied by PANalytical (Almelo, The Netherlands) with Cu Kα1 radiation (λ = 1.54056 Å). The Cu X-ray tube operating at 40 kW/30 mA was used. Diffraction patterns were measured in step-scan mode with a step size of 0.050 and time per step of 10 s. The absorption and emission properties of the synthesized glass samples were examined using optical spectroscopy. The absorption spectra were recorded using a Varian 5000 UV-VIS-NIR spectrophotometer (Cary 5000, Agilent Technology, Santa Clara, CA, USA). Luminescence spectra and their decays were registered using a VIS/NIR laser system. The laser equipment consisted of a Photon Technology International (PTI) Quanta-Master 40 (QM40) UV/VIS Steady State Spectrofluorometer (Photon Technology International, Birmingham, NJ, USA) coupled with a tunable pulsed optical parametric oscillator (OPO), pumped by the third harmonic of a Nd:YAG laser (Opotek Opolette 355 LD, OPOTEK, Carlsband, CA, USA), a 75 W xenon lamp as an excitation source, a double 200 mm monochromator, a multimode UVVIS PMT R928 detector (PTI Model 914), and a Hamamatsu H10330B-75 detector (Hamamatsu, Bridgewater, NJ, USA). The resolution for spectra measurements was ±0.2 nm. Decays were measured with an accuracy of ±2 µs. In order to compare the emission intensity under the same experimental conditions, measurements of glass systems were carried out at the same slit settings. Analysis of the registered data was carried out using OriginPro 9.1 software. All optical measurements were carried out at room temperature.

## 3. Results and Discussion

**Figure 1:**
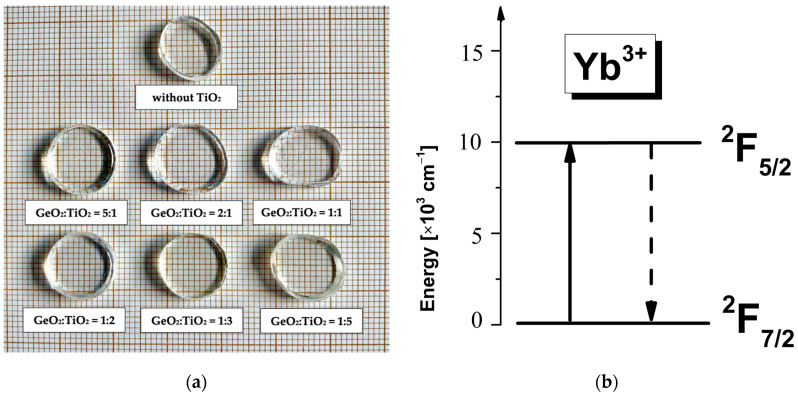
Photographs of the Yb^3+^-doped samples after polishing (**a**) and a simplified energy level diagram of Yb^3+^ (**b**).

Glasses can be prepared in a wide variety of sizes and forms with excellent transparency and homogeneity. In this work, a series of Yb^3+^-doped titanate–germanate glasses in the form of bulk samples were successfully synthesized and their optical properties were examined in detail in this section. Figure 1 shows the obtained Yb^3+^-doped glass samples with various GeO_2_:TiO_2_ molar ratios (a) and an energy level diagram for Yb^3+^ ions (b).

### 3.1. Yb^3+^-Doped Titanate–Germanate Glasses: X-Ray Diffraction Analysis

**Figure 2:**
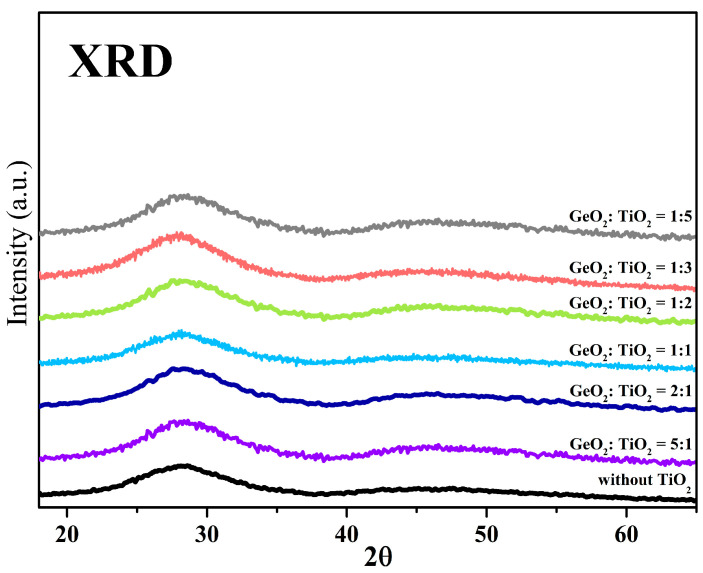
X-ray diffraction patterns of glass without TiO_2_ and titanate–germanate glasses with various GeO_2_:TiO_2_ molar ratios.

As mentioned in the Section 1, the XRD measurements for glasses containing TiO_2_ [11,12] revealed that they are partly crystallized. Figure 2 presents X-ray diffraction patterns of the Yb^3+^-doped titanate–germanate glass samples in the absence and with varying concentrations (from 10 to 50 mol%) of TiO_2_. The observed diffraction patterns showed the amorphous character of all the samples. Importantly, the diffractograms confirmed the absence of narrow diffraction lines typical of crystalline materials. It suggests the absence of the evolution to a lower degree of the order of the local structure and a relatively broad glass-forming region for the studied compositions.

### 3.2. Yb^3+^-Doped Titanate–Germanate Glasses: Effect of TiO_2_ on Near-IR Luminescence Properties

**Figure 3:**
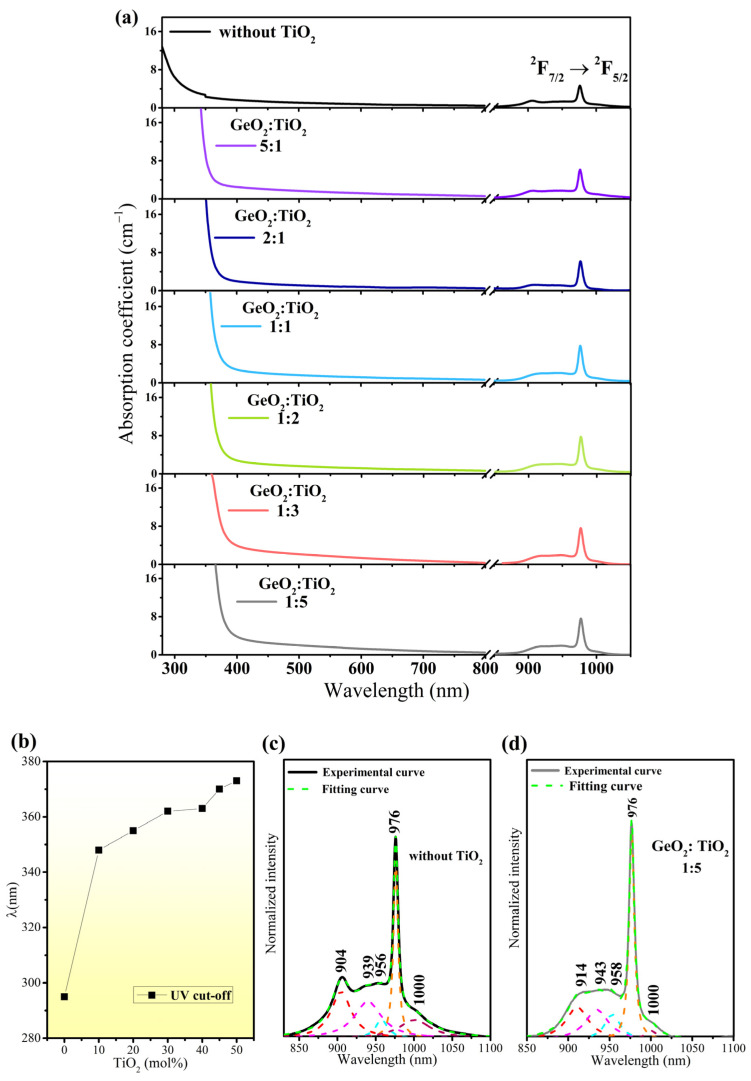
Absorption spectra for Yb^3+^ ions in titanate–germanate glasses with various GeO_2_:TiO_2_ molar ratios compared to GeO_2_-BaO-Ga_2_O_3_ glass (**a**) and UV cut-off (**b**). Deconvolution of the absorption spectra for sample without TiO_2_ (**c**) and sample containing predominantly TiO_2_ (50%mol) (**d**).

The study of the absorption spectra reveals the structural and optical features of the glasses that are suitable for various applications. Figure 3a shows the absorption spectra measurements for Yb^3+^ ions in barium gallo-germanate glasses modified by TiO_2_.

The absorption spectra of fabricated glasses doped with trivalent ytterbium ions were measured in the UV-visible and near-infrared spectral ranges and show that the chemical composition of the glass host has a significant impact on the absorption edge and intensity. For each glass sample, the absorption spectra exhibit the typical absorption band of Yb^3+^ ions, which corresponds to the 4f-4f transition of Yb^3+^ from the ground state (^2^F_7/2_) to the excited state (^2^F_5/2_). The absorption measurements for Yb^3+^ ions clearly indicate that the intensity of this band gradually increases with the increase in the TiO_2_ content. In this study, we used the procedure presented in the work written by Yan et al. [37], which described the deconvoluted absorption spectra for the P_2_O_5_-GeO_2_ glass system in detail. The absorption spectra of Yb^3+^ in barium gallo-germanate glass and titanate–germanate glass containing 50 mol% TiO_2_ were deconvoluted by Lorentzian functions. The deconvolution of the Yb^3+^ ion absorption band showing its Stark splitting is presented in Figure 3c,d. The absorption spectra for selected glass samples are deconvoluted in peaks to resolve the overlapping bands, which originate from the Stark splitting of J manifolds of Yb^3+^ ions. Similar absorption spectra have been registered previously by Z. Yang et al. [38] for Yb^3+^ ions in lead–germanate (10Na_2_O–60GeO_2_–30PbO) systems and indicated that for glasses, due to the low symmetry at Yb^3+^ sites, a clearly resolved band at 976 nm is observed. Similar results were also described by M. Seshardi et al. [39], who conducted a detailed optical study of Yb^3+^-doped borophosphate glasses for IR laser application. Another pointed out that the important issue is the local environment around Yb^3+^. It should be noted that the position of the first deconvoluted peak at 904 nm (sample without TiO_2_) shifts to a longer wavelength (red shift) in the comparison in Figure 3d (sample with 50mol% TiO_2_). A similar effect was observed by Wang et al. [40] in highly Yb-doped silica glass containing various amounts of fluorine doping. Moreover, the effect of TiO_2_ on the optical properties of fabricated Yb^3+^-doped glasses is evidently confirmed by the UV cut-off wavelength. The UV cut-off wavelength, defined as the intersection between the zero baseline and the extrapolation of the absorption edge, was determined. For the sample without TiO_2_, the absorption edge is observed at 295 nm. The UV cut-off (Figure 3b) for titanate–germanate glass is shifted to longer wavelengths with increasing TiO_2_ concentration (389 nm for glass with molar ratio GeO_2_:TiO_2_ = 1:5). These changes can be explained by the modification with the ligand field around Yb^3+^ ions by the incorporation of TiO_2_, which is fully consistent with our previous spectroscopic results for rare-earth-doped titanate–germanate glasses [14,15,16,17,19] and undoped glass hosts [18] for near-infrared luminescence applications.

**Figure 4:**
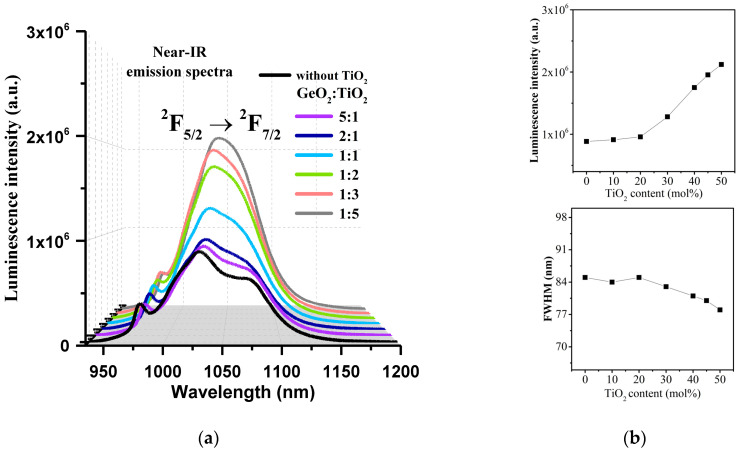
Near-IR luminescence spectra of Yb^3+^ ions in fabricated glass samples (**a**) and emission intensity for the main ^2^F_5/2_ → ^2^F_7/2_ laser transition and emission bandwidth (**b**) as a function of TiO_2_ content.

Figure 4a presents the near-IR emission spectra (λ_exc_ = 980 nm) of Yb^3+^ ions in multicomponent titanate–germanate glasses. The spectra are compared with the sample without TiO_2_.

**Figure 5:**
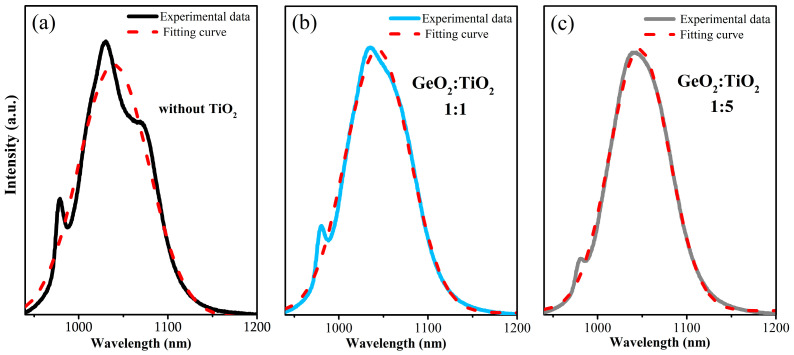
Determination of the FWHM parameter for selected glass samples without TiO_2_ (**a**), and with molar ratio GeO_2_:TiO_2_ = 1:1 (**b**) and 1:5 (**c**) using Gaussian function.

The registered near-IR emission band is related to the main ^2^F_5/2_ → ^2^F_7/2_ laser transition of Yb^3+^. It is clearly seen that the emission spectrum for the Yb^3+^-doped barium gallo-germanate glass sample consists of three emission peaks centered at 980 nm, 1030 nm, and 1070 nm. In general, spectroscopic studies indicate that the intensity of the luminescence band of Yb^3+^ ions is enhanced with increasing titanium dioxide concentration in the GeO_2_-BaO-Ga_2_O_3_ system. Notably, the strongest emission peak is located at 1030 nm, and the intensity begins to increase (2.3-fold increase) when the TiO_2_ concentration is beyond 30 mol% (Figure 4b). On the other hand, the intensity of the emission peak located at about 1070 nm decreases with the increase in TiO_2_ content, and for systems where TiO_2_ acts as a network-former component (TiO_2_ > 30 molar%), it is poorly detectable. In this section, a spectroscopic parameter, such as the full width at half maximum, was estimated for the synthesized glasses, similar to previous work on Yb^3+^ ions in inorganic glasses [24,35,41,42] and other lanthanides [36,43,44,45,46,47,48]. In general, the luminescence bandwidth, referring to the FWHM for the main ^2^F_5/2_ → ^2^F_7/2_ laser transition at 1030 nm of Yb^3+^, is nearly independent on TiO_2_ concentration, and its value is close to 83 nm (Figure 4b). The results of calculating the full width at half maximum for the main ^2^F_5/2_ → ^2^F_7/2_ laser transition of Yb^3+^ ions using the Gaussian function in OriginPro software are presented in Figure 5. A fitting procedure was also used in the study presented by the research group of Boulon et al. for Yb^3+^-doped lead silicate glasses [49], Yb^3+^-doped silica fibers [50], and also for Yb^3+^ ions in tellurite glass [51] or Yb^3+^ ions in lead phosphate glasses [52].

The observed optical emission spectra of Yb^3+^ ions in the titanate–germanate glass is very similar to the corresponding Yb^3+^ optical spectra observed in lead fluoroborate glass [53], and completely different than Yb^3+^-doped glass-ceramics [54] or ytterbium(III) complexes [55,56]. N. Purnachand [57] and co-workers analyzed the properties of Yb^3+^-doped lead antimony borate glass belonging to the high-phonon glass family and registered the emission spectra of the studied samples. It was observed that the PbO–Sb_2_O_3_–B_2_O_3_ system exhibited a stronger emission band at about 978 than at 1014 nm due to the ^2^F_5/2_ → ^2^F_7/2_ transition. Similar results were also described by D. Rajeswara Rao et al. [58], who conducted a detailed optical study of Yb^3+^ ions in a PbO–PbF_2_–B_2_O_3_ glass system. Studies of glasses doped with Yb^3+^ ions indicated these systems could emit a near-IR luminescence band as a result, and its intensity strongly depends on the glass host, chemical composition, network-former oxide, network-modifier oxide, and/or Yb^3+^ ion concentration. Moreover, the enhanced and broad emission spectrum obtained for multicomponent glass with Yb^3+^ ions with a molar ratio of GeO_2_:TiO_2_ = 1:5 (50 mol% TiO_2_) shows its promise as an attractive medium operating over a wide range of NIR wavelengths (1–1.2 μm).

**Figure 6:**
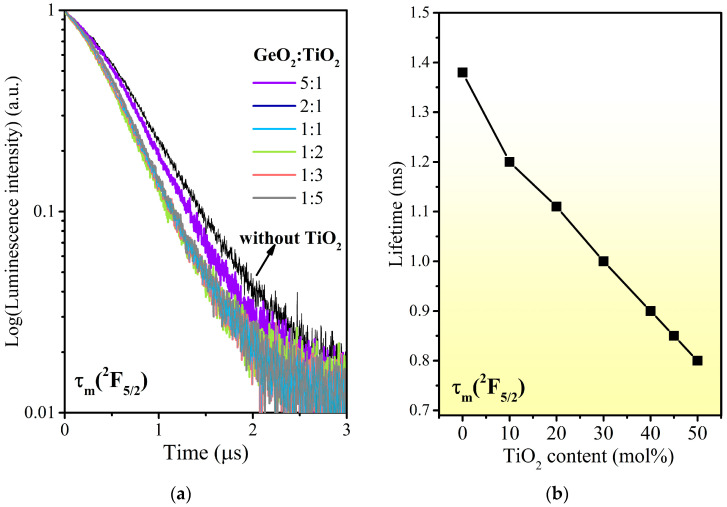
Luminescence decay curves of the ^2^F_5/2_ state for the investigated glass samples doped with Yb^3+^ ions with various GeO_2_:TiO_2_ molar ratios (**a**) and luminescence lifetimes (**b**).

For further investigation, luminescence decays from the ^2^F_5/2_ state of Yb^3+^ ions have been analyzed in detail. The decay curves for the ^2^F_5/2_ (Yb^3+^) state in multicomponent titanate–germanate glasses were measured under excitation at 980 nm and while monitoring the emission wavelength at 1030 nm. The luminescence decay curves for Yb^3+^ are presented in Figure 6. The obtained results clearly demonstrated that decays are reduced with increasing TiO_2_ concentration in the glass composition. Based on decay curve measurements, luminescence lifetimes for the ^2^F_5/2_ state of the Yb^3+^ ion level were evaluated from a nearly single exponential fit. The measured lifetimes for the ^2^F_5/2_ state of Yb^3+^ varying with TiO_2_ concentration are schematically shown in Figure 6b.

The longest lifetime measured here is 1.38 ms for the Yb^3+^-doped barium gallo-germanate glass, which is longer than that reported for the Yb^3+^-doped Ga_2_O_3_-GeO_2_-BaO-K_2_O system (0.95 ms) [59]. It is observed that the ^2^F_5/2_ excited state decreases from 1.21 ms to 0.80 ms in the investigated glasses when TiO_2_ concentration increases from 10% to 50 mol%. This decrease was related to the concentration quenching effect. Concentration quenching has been analyzed for different optical matrices containing Yb^3+^ ions. This phenomenon was also described by P. Yang et al. [60], who conducted a detailed optical study of Yb:YAG crystals with a high Yb^3+^ doping level. Kummara et al. [61] determined the luminescence lifetime for Yb^3+^-doped oxyfluoride glasses, ranging from 1.52 to 0.18 ms, and showed that the quenching of luminescence lifetime could be due to multiphoton relaxation, direct coupling with OH^−^ groups, or energy transfer among Yb^3+^ ions. In the present study, the Yb^3+^-doped glass samples were prepared under rigorous technological conditions in glove-box, in a protective atmosphere of dried argon of high purity. Additionally, in our previous work for active oxide [62] and oxyfluoride [63] titanate–germanate glasses for near- and mid-IR luminescence applications, extremely low-IR absorption coefficients were achieved. Consequently, it is assumed that the concentration quenching effect connected with the dominant mechanisms of lifetime quenching is multiphoton relaxation as well as energy transfer among the Yb^3+^ ions. The luminescence decay measurements of fabricated glasses for photonic applications confirmed that the analysis of the influence of the GeO_2_:TiO_2_ ratio on spectroscopic parameters of Yb^3+^ ions is crucial in the presented preliminary study. The luminescence lifetime for the excitation state transition is compared to several Yb^3+^-doped luminescent materials published previously. The results are summarized in Table 2.

In general, TiO_2_ is making a great contribution to the field of engineering materials thanks to its unique properties [76,77,78,79,80]. In 2019, X. Kang et al. [81] presented an excellent review highlighting the role of TiO_2_ in engineering and applications. Encouragingly, the glass system containing TiO_2_ is dedicated to acting as an active optical center for rare-earth ions. Importantly, titanium plays a dual role in the glass structure: as the network former or as the network modifier. TiO_2_, a member of 3D transition metal oxides, is considered a valuable glass constituent in some special glasses. Notably, the Judd–Ofelt analysis for Nd^3+^, Er^3+^, Pr^3+^, Tm^3+^, and Ho^3+^ presented in our previous works [14,15,17,19] clearly indicated that TiO_2_ content modifies the degree of covalency of the rare-earth ligand field and fabricated glass systems exhibited more rigidity. A similar relationship between the incorporation of TiO_2_ and GeO_2_ and the properties of telluride glasses was described by Tang et al. [82]. Although the perspectives of germanate-based glasses for photonic applications are very motivating, obtaining glasses with an amorphous nature and enhanced near-infrared luminescence properties is not trivial. Finally, the research in this paper perfectly demonstrates that the use of the TiO_2_ component in a Yb^3+^-doped barium gallo-germanate glass system, which plays a dual role in the glass structure depending on its concentration but presents unique luminescence properties in the near-infrared range, may be an attractive way of designing new optical materials for laser technology. Our preliminary results presented in this paper are in keeping with current standards and also have important implications for the future of photonic glasses. Several spectroscopic and near-infrared (NIR) laser parameters for Yb^3+^ based on theoretical calculations will be discussed in another paper in detail. Certainly, the influence of TiO_2_ concentration on the spectroscopic properties of Yb^3+^ ions needs further study.

## 4. Conclusions

Novel Yb^3+^-doped low-phonon titanate–germanate glasses were developed to understand the impact of TiO_2_ addition on the optical near-infrared luminescence properties. In this study, the preliminary experimental results for Yb^3+^ have been discussed for samples without TiO_2_ and for samples where the GeO_2_:TiO_2_ molar ratio was changed from 5:1 (50 mol% GeO_2_; 10mol% TiO_2_) to 1:5 (10 mol% GeO_2_; 50mol% TiO_2_). We have encountered interesting results that point to the application of TiO_2_-GeO_2_-BaO-Ga_2_O_3_-Yb_2_O_3_ glass for near-infrared photonic applications. The samples where GeO_2_ was substituted by TiO_2_ were examined using X-ray diffraction (XRD), absorption, and luminescence spectroscopies. Based on data analysis, we have shown the following conclusions:

- X-ray diffraction analysis revealed that all received samples are fully amorphous. The absorption edge for Yb^3+^-doped titanate–germanate glass was shifted to longer wavelengths (red shift) with increasing TiO_2_ concentration. The absorption measurements for Yb^3+^ ions clearly indicated that the intensity of the absorption band corresponding to the transition originating from the ^2^F_7/2_ ground state to the ^2^F_5/2_ higher-lying state is the most intense for samples when TiO_2_ plays the role as glass network former.
- Near-IR emission spectra and their decays were analyzed in detail. Adding titanium dioxide into Yb^3+^-doped TiO_2_-GeO_2_-BaO-Ga_2_O_3_ glass significantly influenced the 1 μm luminescent properties. For titanate–germanate glass containing 50%mol TiO_2_, the integrated emission intensity for the main ^2^F_5/2_ → ^2^F_7/2_ laser transition of Yb^3+^ was enhanced more significantly (2.3-fold increase) compared to the sample without TiO_2_. Luminescence decays from the ^2^F_5/2_ state of Yb^3+^ ions were measured. The ^2^F_5/2_ measured lifetimes depend critically on titanium dioxide concentration.

From an application point of view, this titanate–germanate glass, when TiO_2_ plays the role of a glass network former, can be a promising starting material for solid-state lasers. The development of efficient new Yb^3+^-doped titanate–germanate glass will continue to be a research priority for our group in the near future.

## Figures and Tables

**Table 1:** Nominal chemical compositions for Yb^3+^-doped barium gallo-germanate glass samples with various GeO_2_:TiO_2_ molar ratios.

No	Nominal Chemical Composition [mol%]	TiO_2_:GeO_2_ Molar Ratio
1	60GeO_2_-30BaO-9.5Ga_2_O_3_-0.5Yb_2_O_3_	without TiO_2_
2	50GeO_2_-10TiO_2_-30BaO-9.5Ga_2_O_3_-0.5Yb_2_O_3_	5:1
3	40GeO_2_-20TiO_2_-30BaO-9.5Ga_2_O_3_-0.5Yb_2_O_3_	2:1
4	30GeO_2_-30TiO_2_-30BaO-9.5Ga_2_O_3_-0.5Yb_2_O_3_	1:1
5	20GeO_2_-20TiO_2_-30BaO-9.5Ga_2_O_3_-0.5Yb_2_O_3_	1:2
6	15GeO_2_-15TiO_2_-30BaO-9.5Ga_2_O_3_-0.5Yb_2_O_3_	1:3
7	10GeO_2_-50TiO_2_-30BaO-9.5Ga_2_O_3_-0.5Yb_2_O_3_	1:5

**Table 2:** Comparative analysis of luminescence lifetimes in different luminescent materials (glasses, crystals, glass-ceramics) doped with Yb^3+^ ions emitting in the near-infrared range.

Luminescent Material	Luminescence Lifetime [ms]	Reference
GeO_2_-BaO-Ga_2_O_3_-0.5%Yb_2_O_3_	1.38	this work
TiO_2_-GeO_2_-BaO-Ga_2_O_3_-0.5%Yb_2_O_3_ (TiO_2_:GeO_2_ = 1:5)	1.21	this work
TiO_2_-GeO_2_-BaO-Ga_2_O_3_-0.5%Yb_2_O_3_ (TiO_2_:GeO_2_ = 1:1)	1.02	this work
TiO_2_-GeO_2_-BaO-Ga_2_O_3_-0.5%Yb_2_O_3_ (TiO_2_:GeO_2_ = 5:1)	0.80	this work
Ga_2_O_3_-GeO_2_-BaO-K_2_O:0.5%Yb_2_O_3_	0.97	[59]
Li_2_B_4_O_7_:0.4%Yb	0.48	[64]
MgF_2_-BaF_2_-Al(PO_3_)_3_-Ba(PO_3_)_2_:1%Yb_2_O_3_	0.65	[65]
Na(PO_3_)_3_-Al(PO_3_)_3_:1%Yb_2_O_3_	0.90	[66]
Al_2_O_3_-SiO_2_:0.3%Yb_2_O_3_	0.92	[67]
P_2_O_5_-K_2_O-MgO-Al_2_O_3_-0.5%Yb_2_O_3_	0.96	[68]
GeO_2_-PbO-Ga_2_O_3_-Na_2_O:1.5%Yb_2_O_3_	1.76	[69]
NaPO_3_-Na_2_O-NaF-1.25%Yb_2_O_3_	1.8	[70]
ZrO_2_-1%Yb^3+^:Y_2_O_3_	1.85	[71]
P_2_O_5_-ZnO-Al_2_O_3_-BaO-PbO-1.0%Yb_2_O_3_	1.45	[72]
CaF_2_:1%Yb^3+^	2.88	[73]
P_2_O_5_-B_2_O_3_-Li_2_O-ZnO:0.5%Yb_2_O_3_	1.73	[74]
Bi_2_O_3_-B_2_O_3_:2%Yb_2_O_3_	0.34	[75]

## Data Availability

The original contributions presented in this study are included in the article. Further inquiries can be directed to the corresponding authors.
